# Inhibition of autophagy, lysosome and VCP function impairs stress granule assembly

**DOI:** 10.1038/cdd.2014.103

**Published:** 2014-07-18

**Authors:** S J Seguin, F F Morelli, J Vinet, D Amore, S De Biasi, A Poletti, D C Rubinsztein, S Carra

**Affiliations:** 1 Dipartimento di Scienze Biomediche, Metaboliche e Neuroscienze, Universita' di Modena e Reggio Emilia, Modena, Italy; 2 Dipartimento Chirurgico, Medico, Odontoiatrico e di Scienze Morfologiche, Universita' di Modena e Reggio Emilia, Modena, Italy; 3 Dipartimento di Scienze Farmacologiche e Biomolecolari (DiSFeB), Universita' di Milano, Milan, Italy; 4 Department of Medical Genetics, Cambridge Institute for Medical Research, University of Cambridge, Hills Road, Cambridge, UK

## Abstract

Stress granules (SGs) are mRNA-protein aggregates induced during stress, which accumulate in many neurodegenerative diseases. Previously, the autophagy-lysosome pathway and valosin-containing protein (VCP), key players of the protein quality control (PQC), were shown to regulate SG degradation. This is consistent with the idea that PQC may survey and/or assist SG dynamics. However, despite these observations, it is currently unknown whether the PQC actively participates in SG assembly. Here, we describe that inhibition of autophagy, lysosomes and VCP causes defective SG formation after induction. Silencing the VCP co-factors UFD1L and PLAA, which degrade defective ribosomal products (DRIPs) and 60S ribosomes, also impaired SG assembly. Intriguingly, DRIPs and 60S, which are released from disassembling polysomes and are normally excluded from SGs, were significantly retained within SGs in cells with impaired autophagy, lysosome or VCP function. Our results suggest that deregulated autophagy, lysosomal or VCP activities, which occur in several neurodegenerative (VCP-associated) diseases, may alter SG morphology and composition.

Cells respond to stresses, like heat shock or oxidative agents, which lead to protein aggregation, by activating the protein quality control (PQC) and attenuating translation.^1^ The PQC consists of molecular chaperones and degradation systems and is an essential player of the proteotoxic stress response. To minimize protein aggregation, chaperones assist protein folding; when this is not effective, chaperones assist in targeting damaged substrates for clearance by the ubiquitin–proteasome system (UPS) and the lysosome-based degradation systems.^2, 3^ In parallel, polysomes disassemble, releasing ribosomes, mRNAs, defective ribosomal products (DRIPs) and newly synthesized proteins, which, due to the stress, are prone to aggregation and are subjected to PQC and degradation.^4^

The mRNAs encoding ‘housekeeping' proteins released from disassembling polysomes are sequestered into stress granules (SGs), non-membranous cytoplasmic foci where mRNAs are stored during stress.^5^ SGs have heterogeneous compositions and contain translationally silent mRNAs, early initiation factors, small, but not large, ribosomal subunits, mRNA-binding proteins, kinases and signaling molecules.^5^ Selective sequestration of these components within SGs occurs in a challenging subcellular environment where aggregate-prone substrates (released by polysomes) tend to accumulate. SG assembly is also triggered by the self-aggregation of RNA-binding proteins that contain prion-like domains, including T-cell-restricted intracellular antigen-1 (TIA-1).^6^ Unlike prionogenic fibrillar aggregates, SGs are dynamic structures, which disassemble within few hours after their formation.

Due to the heterogeneous composition of SGs and to the crowded molecular environment, SGs may, indirectly, require PQC assistance for proper assembly and disassembly. A number of SG components have a role in PQC, including ubiquitin and E3 ubiquitin ligases (TNF receptor-associated factor 2 and Roquin),^7, 8, 9, 10^ while proteasome inhibition induces SGs.^11^ Histone deacetylase 6 (HDAC6), another SG component,^8^ facilitates the clearance of misfolded ubiquitinated proteins and participates in their targeting to the aggresome, a perinuclear structure that forms in response to an overload of un/misfolded proteins and enhances the degradation of toxic proteins.^12^ Moreover, HDAC6 binds to another SG component, Ras-GTPase-activating protein SH3 domain-binding protein (G3BP), which modulates the de-ubiquitinating enzyme ubiquitin specific peptidase 10 (USP10), which is also required for SG formation.^13, 14^ Although the exact role of these PQC components in SG dynamics is only partly understood, these findings suggest that PQC and SGs are interconnected systems. SGs are degraded via macroautophagy (which we call autophagy) via a mechanism requiring the ubiquitin-selective chaperone valosin-containing protein (VCP).^15^ VCP modulates the ubiquitin-dependent proteolysis of selective clients by proteasome, ER-associated degradation and/or autophagosomes;^16, 17, 18^ this underscores the link between SGs and proteostasis. Here, we investigated whether impairment of PQC, autophagy and lysosomes affects SG assembly. We demonstrate that inhibition of VCP, autophagy or lysosomes affects SG formation, morphology and composition.

## Results

### Lysosomal inhibition impairs SG formation

**Figure 1:**
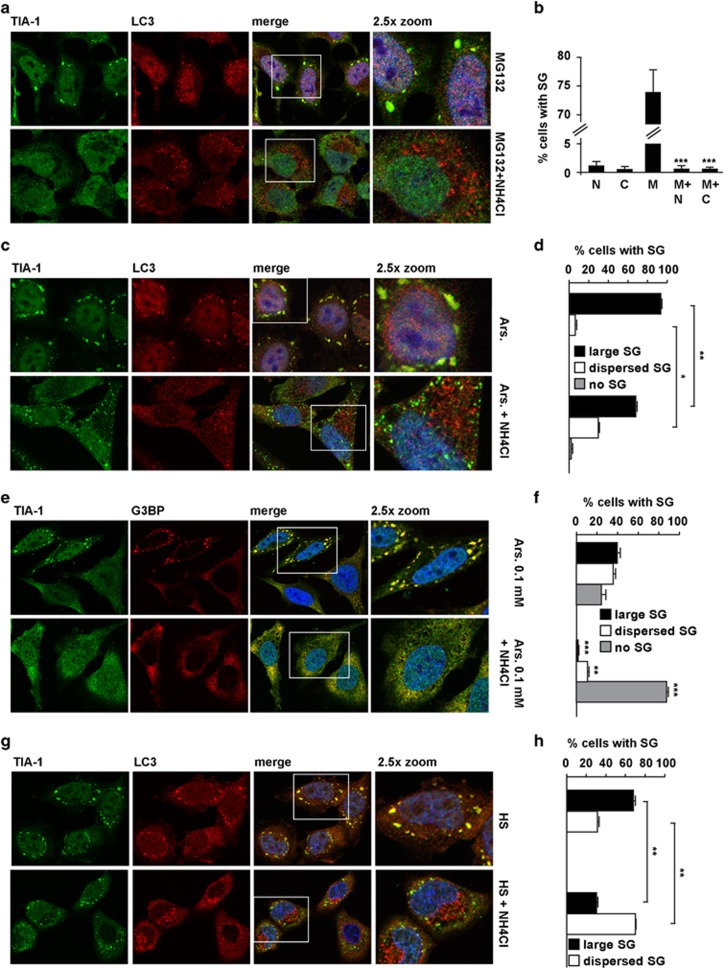
Lysosomotropic agents severely impair SG formation. HeLa cells treated for 3 h with MG132 alone or with ammonium chloride (NH_4_Cl; **a**, **b**) or chloroquine (CLQ; **b**) were fixed and labeled with anti-TIA-1, LC3 and DAPI. (**b**) Percentage of cells with TIA-1-positive SGs is shown (M=MG132; N=NH_4_Cl; C=CLQ). Error bar, S.E.M. ***P<0.001 compared with MG132. HeLa cells were treated for 45 min with 0.5 mM (**c**, **d**) or 0.1 mM (**e**, **f**) arsenite (Ars.); where indicated, cells were pretreated for 2 h 15 min with ammonium chloride (NH_4_Cl). Cells were fixed and labeled with anti-TIA-1, LC3 (**c**) or G3BP (**e**) and DAPI. (**d**, **f**) Percentage of cells with TIA-1-positive SGs (large or dispersed) and no SGs is shown. Error bar, S.E.M. (**e**) *P<0.05; **P<0.01; (**f**) ***P<0.001; **P<0.01 compared with Ars. 0.1 mM. (**g**, **h**) Hela cells pretreated or not with ammonium chloride (NH_4_Cl) for 2 h 15 min were subjected to heat shock (HS) at 43.5 °C for 45 min, fixed and labeled with anti-TIA-1, LC3 and DAPI. (**h**) Quantitation of data in **g**. Error bar, S.E.M. **P<0.01. (**a, c, e, g**) 2.5 × magnification of the selected area. See also Supplementary Figures S1, S2 and S3

To assess whether impairment of the PQC system may affect SG formation, we induced SGs using the proteasome inhibitor MG132^11^ and simultaneously inhibited the lysosomes, the common endpoint for a number of degradation pathways.^19, 20^ In line with previous reports,^11^ MG132 induced SGs in ca 70% of the cells, with a maximal peak after 3 h; SGs, which are dynamic, disappeared after 8 h of MG132 treatment (Supplementary Figure S1A). Co-treatment of the cells with MG132 and the lysosomal inhibitors ammonium chloride (NH_4_Cl) or chloroquine (CLQ) suppressed MG132-induced SGs (Figures 1a and b). We saw no SGs even after 8 h co-treatment with MG132 and NH_4_Cl (Supplementary Figure S1A), which supports an inhibitory role, rather than a delay, on SG formation. MG132 caused the accumulation of ubiquitinated proteins, while NH_4_Cl increased levels of LC3-II, the autophagosome-associated, lipidated form of LC3 that accumulates if their lysosomal degradation is inhibited^21^ (Supplementary Figure S1A). When we monitored SGs and the efficacy of NH_4_Cl, which causes a buildup of LC3-positive autophagosomes, by immunofluorescence using antibodies for TIA-1 and LC3, respectively, we observed LC3 recruitment to SGs (Figure 1a).

Since drugs that stabilize polysomes (cycloheximide) inhibit SG formation,^22^ we analyzed polysome distribution to test whether NH_4_Cl prevented polysome disassembly. NH_4_Cl did not alter the distribution of ribosomal protein S6 (RPS6), while addition of EDTA to pelleted polysomes dissociated them, as expected (Supplementary Figures S1B and C). MG132 alone, or with NH_4_Cl, caused polysome disassembly, as no or only a minor RPS6 signal was detected throughout the gradient (Supplementary Figure S1C). Thus, NH_4_Cl prevents MG132-induced SGs by acting downstream of polysome disassembly.

Next, we tested if NH_4_Cl inhibited SG formation in response to arsenite, which also leads to protein aggregation.^23^ Arsenite (0.5 mM) induced large SGs aligned in a ring-like shape in nearly all the cells (number of SGs/cell: 10.5±0.3; SG size: 2.03 *μ*M±0.05; Figures 1c and d). Pre-treatment with NH_4_Cl modified the number, size and distribution of SGs, which were more numerous, smaller (number of SGs/cell: 24.6±0.7; SG size: 0.94 *μ*M±0.02) and dispersed throughout the cytoplasm (Figures 1c and d). NH_4_Cl abrogated SG formation when we used lower concentrations of arsenite (0.1 mM), which did not lead to a maximal SG response (Figures 1e and f).

SGs form as a consequence of translation arrest (but are not required for translation arrest).^24^ Translational status was assessed using puromycin, which is incorporated into nascent peptides.^25^ Puromycin-labeled proteins were detected in control cells and cells treated with NH_4_Cl, but not after treatment with arsenite alone or combined with NH_4_Cl (Supplementary Figure S1D). In parallel, polysomes disassembled after arsenite and NH_4_Cl co-treatment (data not shown).

Assembly of SGs is triggered by the self-reversible aggregation of components that contain a prion-like domain, including TIA-1.^6^ We excluded that the small and dispersed structures that form in cells co-treated with arsenite and NH_4_Cl are proteinaceous aggregates rather than bona fide SGs, since both G3BP, a well-known SG marker (Figure 1e), and RNA, labeled using SYTO RNASelect (Supplementary Figure S2A), colocalized with TIA-1 in these cells.

**Figure 2:**
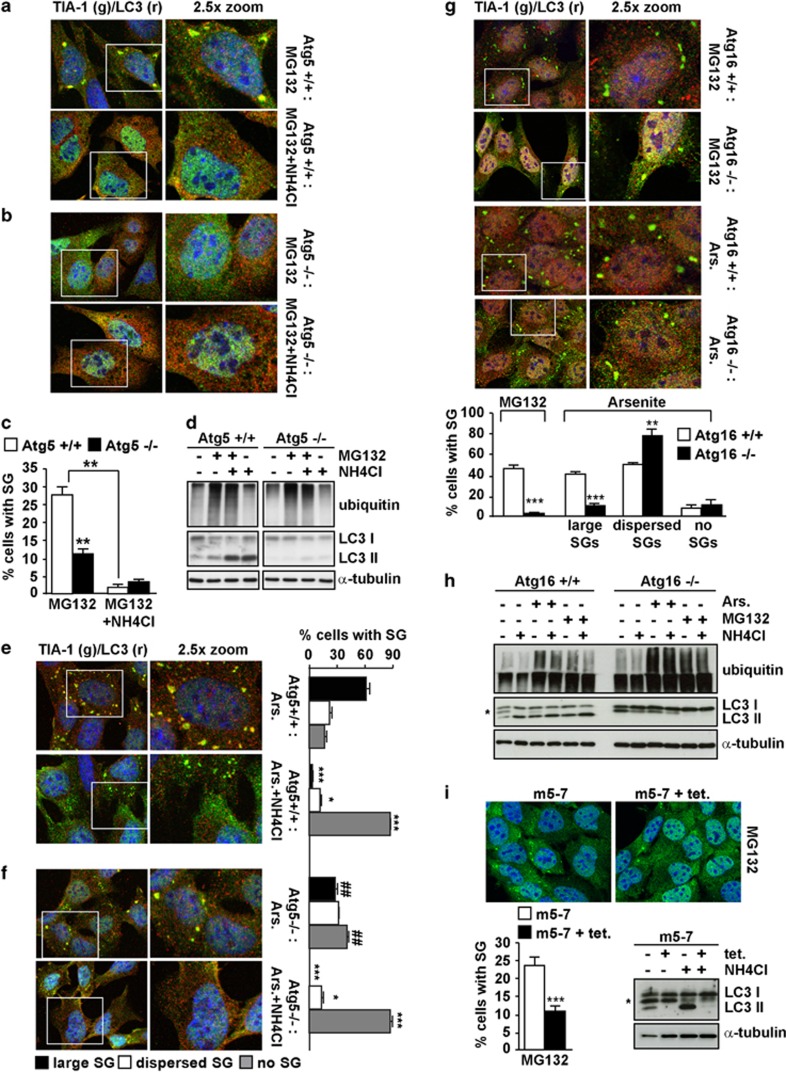
Atg5 and Atg16 null cells show impaired SG formation. (**a–d**) Atg5^+/+^ or Atg5^−/−^ MEFs were left untreated or treated for 3 h with MG132 and/or ammonium chloride (NH_4_Cl) and either fixed and labeled with anti-TIA-1, LC3 and DAPI (**a–c**) or processed for western blot (**d**). (**c**) Quantitation of data in **a** and **b**. Error bar, S.E.M. **P<0.01 compared with MG132 in Atg5^+/+^. (**e, f**) Atg5^+/+^ and Atg5^−/−^ MEFs were treated for 45 min with arsenite (Ars.); where indicated, MEFs were pretreated for 2 h 15 min with 20 mM ammonium chloride (NH_4_Cl). Cells were fixed and labeled with anti-TIA-1, LC3 and DAPI. Quantitation of data is shown. Error bar, S.E.M. ***P<0.001; *P<0.05 compared with Ars.; ^##^*P*<0.01 Ars-treated Atg5^−/−^ compared with Atg5^+/+^. (**g**) Atg16^+/+^ and Atg16^−/−^ MEFs were treated for 3 h with MG132 or for 45 min with Ars., fixed and labeled with anti-TIA-1, LC3 and DAPI. Quantitation of data is shown. Error bar, S.E.M. ***P<0.001; **P<0.01. (**h**) Atg16^+/+^ and Atg16^−/−^ MEFs were treated as described in **a**; where indicated cells were pretreated for 2 h 15 min with ammonium chloride (NH_4_Cl), prior to lysis and western blot. (**i**) m5-7 cells were grown for 7 days without (−) or with (+) tetracycline (500 ng/ml), prior to addition of ammonium chloride (NH_4_Cl) or MG132 for 3 h. Cells were processed for western blot or fixed and labeled with anti-TIA-1 and DAPI. Quantitation of data is shown. Error bar, S.E.M. ***P<0.001 compared with MG132 condition. (**h**, **i**) * likely corresponds to LC3 T. (**a, b, e–g**) 2.5 × magnification of the selected area

To generalize the inhibitory effect of NH_4_Cl on SGs, we induced SGs with heat shock; NH_4_Cl also decreased the formation of heat shock granules (Figures 1g and h). Then, to further test the importance of lysosomes in SG formation, we used different combinations of lysosomal protease inhibitors leupeptin, E64d and pepstatin A. While NH_4_Cl and CLQ inhibit the activity of all lysosomal acid hydrolases, these inhibitors selectively target the activity of specific classes of proteases. Pre-treatment with leupeptin and E64d or leupeptin, E64d and pepstatin A caused both LC3-II accumulation and reduced MG132-induced SGs (Supplementary Figure S2B). LC3 recruitment into SGs was found under all stress conditions tested and was confirmed using three LC3 antibodies (Supplementary Figure S2C; Figures 1a, c and g). To determine which form of LC3, cytosolic LC3-I or autophagosome-anchored LC3-II, is recruited into SGs, we used Atg5 (autophagy gene 5) knockout (−/−) MEFs; these cells lack the autophagy ATG5 gene required for LC3-I to LC3-II conversion (Figure 2d).^26^ Also in Atg5^−/−^ MEFs, SGs were positive for LC3, suggesting that it is cytosolic LC3-I that is recruited into SGs and consistent with SGs being non-membranous foci (Supplementary Figure S2D). NH_4_Cl impaired SG formation in Atg5^−/−^ and Atg5^+/+^ MEFs, generalizing its inhibitory effect to different cell lines (Figures 2a–f). After MG132 or arsenite treatments, the percentage of cells positive for SGs was significantly reduced in Atg5^−/−^ MEFs, as compared with Atg5^+/+^ MEFs (Figures 2a–f), thereby suggesting a potential interplay between autophagy and SGs. We further tested this hypothesis by using MEFs lacking the ATG16 gene, which is essential for autophagosome formation,^27^ as evidenced by the lack of LC3-II in Atg16^−/−^ cells (Figures 2g and h). Similar to Atg5^−/−^ cells, Atg16^−/−^ cells showed impaired SG formation following MG132 or arsenite treatments (Figure 2g). However, NH_4_Cl has an additional inhibitory effect on SGs in Atg5^−/−^ cells (Figure 2f), suggesting that other macroautophagy-independent routes may contribute to SG formation.^28^ Finally, to confirm whether autophagy influences SG assembly, we used modified Atg5^−/−^ MEFs that express an ATG5 transgene which is negatively regulated by tetracycline (m5-7), allowing inducible suppression of autophagy in the same cell line.^29^ Addition of tetracycline to the m5-7 cells suppressed LC3 lipidation (Figure 2i). MG132 treatment induced SGs in ca 25–30% of autophagy-competent m5-7 cells grown in the absence of tetracycline, similar to what was observed in Atg5^+/+^ MEFs (Figure 2, compare c and i). Addition of tetracycline to these m5-7 cells, which switches off autophagy/ATG5 expression, impaired MG132-induced SGs (Figure 2, compare c and i). Thus, inhibition of autophagy and lysosomes impairs SG assembly. This is complementary to recent findings demonstrating that SGs are cleared by autolysosomes and accumulate during the recovery, if autophagy is inhibited^15^ (a phenomenon we confirmed; Supplementary Figure S3A).

While NH_4_Cl (and CLQ) abrogated SG formation induced by MG132, they severely impaired (but did not block) SG formation by arsenite (0.5 mM) or heat shock. We thus hypothesized that interplay between proteasome and lysosomes may exist and modulate SG assembly. To address this hypothesis, we treated HeLa cells with MG132 and arsenite (0.5 mM), which together induce large SGs in all cells (Supplementary Figures S3B and C). Addition of NH_4_Cl inhibited SG assembly (Supplementary Figures S3B and C). Next, we used a low dose of Bortezomib, which inhibits the proteasome but does not induce SGs (Supplementary Figures S3D and E). Overnight pre-treatment with Bortezomib inhibited arsenite-induced SGs (Supplementary Figures S3D and E). These results support that interplay between proteasome and lysosomes is required for optimal SG assembly.

We thus asked how inhibition of the main protein degradation pathways may impair SG formation. While the 70 kd heat shock protein (Hsp70) overexpression inhibits SG formation,^11^ we did not observe major induction of Hsp70 after short-term co-treatments with MG132 or arsenite and NH_4_Cl (Supplementary Figure S3F), or with overnight treatment with leupeptin, E64d and pepstatin A (Supplementary Figure S3G). Upregulation of Hsp70 was only found after overnight treatment with bortezomib (Supplementary Figure S3G). Thus, SG formation is not impaired by Hsp70 upregulation in our experimental conditions. Phosphorylation of the alpha subunit of translation initiation factor 2 (eIF2*α*) is required for the induction of SGs upon arsenite, but not MG132.^30^ Arsenite alone or in combination with NH_4_Cl induced eIF2*α* phosphorylation (Supplementary Figure S3H), pointing to a different mechanism responsible for impaired SG assembly upon lysosome inhibition. We excluded the possibility that apoptosis may account for our observations, since pre-treatment with the pan-caspase inhibitor zVAD-fmk could not rescue the inhibitory effect exerted by NH_4_Cl on MG132- or arsenite-induced SGs (Supplementary Figure S3I), and the percentage of apoptotic and necrotic cells and cells with depolarized mitochondria were similar under all conditions tested both in HeLa (Supplementary Table S1) and autophagy-proficient/deficient cells (Supplementary Table S2).

### Depletion of VCP impairs SG formation

**Figure 3:**
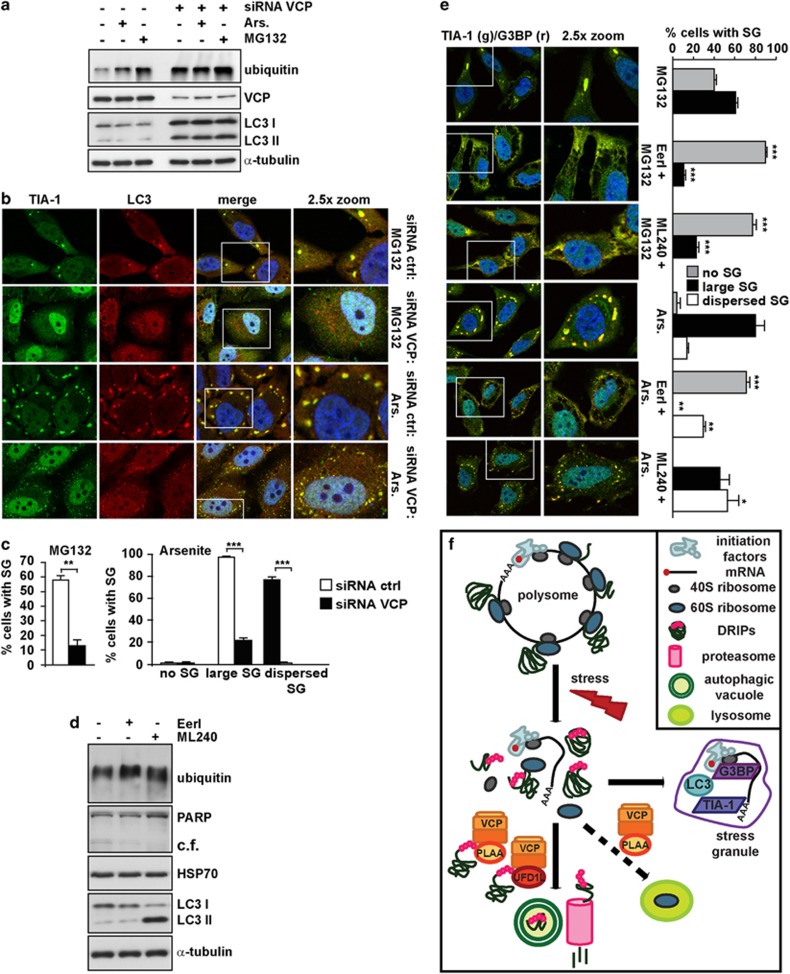
Depletion and inhibition of VCP severely impair SG formation. (**a–c**) HeLa cells lipofected with control (ctrl) or VCP siRNA for 72 h were left untreated (data not shown) or treated for 3 h with MG132 or 45 min with arsenite (Ars.) Cells were lysed, processed for western blot (**a**) or fixed and labeled with anti-TIA-1, LC3 and DAPI (**b**). (**c**) Quantitation of data in **b**. Error bar, S.E.M. **P<0.01; ***P<0.001. (**d**) HeLa cells not treated or treated for 3 h with ML240 or EerI were lysed and processed for ubiquitin, PARP (c.f.: cleaved-fragment), Hsp70, LC3 and α-tubulin western blot. (**e**) Cells were treated for 3 h with MG132 or for 45 min with Ars. alone or in combination with ML240 or EerI; cells were fixed and labeled with anti-TIA-1, G3BP and DAPI. Quantitation of data is shown. Error bar, S.E.M. For MG132 co-treatments: ***P<0.001 compared with MG132 alone; for Ars. co-treatments: ***P<0.001; **P<0.01; *P<0.05 compared with Ars. alone. (**f**) Schematic model of the known role of VCP in targeting DRIPs and large ribosome subunits (60S) to degradation. Polysomes are a cluster of ribosomes that synthesize nascent chains from mRNAs. Upon stress, including MG132 or arsenite treatments, polysomes disassemble and their components follow different fates. mRNAs together with initiation factors and the small (40S) ribosome subunit are sequestered into stress granules (SGs). DRIPs are ubiquitinated and routed to degradation. VCP bound to 60S modulates the degradation of DRIPs. Co-factors such as UFD1L and PLAA assist VCP in client targeting to both proteasome and autophagy for clearance. In parallel, 60S are also released but are excluded from SGs and can become damaged under stress conditions (e.g., oxidation due to arsenite). 60S are targeted to lysosome for degradation, a process called ribophagy. VCP and PLAA are also required in yeast for ribophagy. (**b**, **e**, **f**) 2.5 × magnification of the selected area. See also Supplementary Figure S4

VCP knockdown in HeLa cells led to accumulation of ubiquitinated proteins and LC3-II (Figure 3a)^17^ and decreased SG formation induced by MG132 and arsenite (Figures 3b and c). The arsenite-induced SGs forming in VCP-deficient cells also contained LC3 (Figure 3b), supporting that LC3 recruitment within SGs is independent on VCP. Two well-known inhibitors of VCP, Eeyarestatin I (EerI) and ML240 led to a mild accumulation of ubiquitinated proteins, while only ML240 also caused LC3-II buildup, in line with previous findings (Figure 3d);^31^ however, they did not induce poly ADP ribose polymerase (PARP) cleavage or Hsp70 (Figure 3d). Neither EerI nor ML240 induced SGs (data not shown); instead, they impaired SG assembly following MG132 or arsenite treatment (Figure 3e), consistent with what we observed with VCP knockdown.

**Figure 4:**
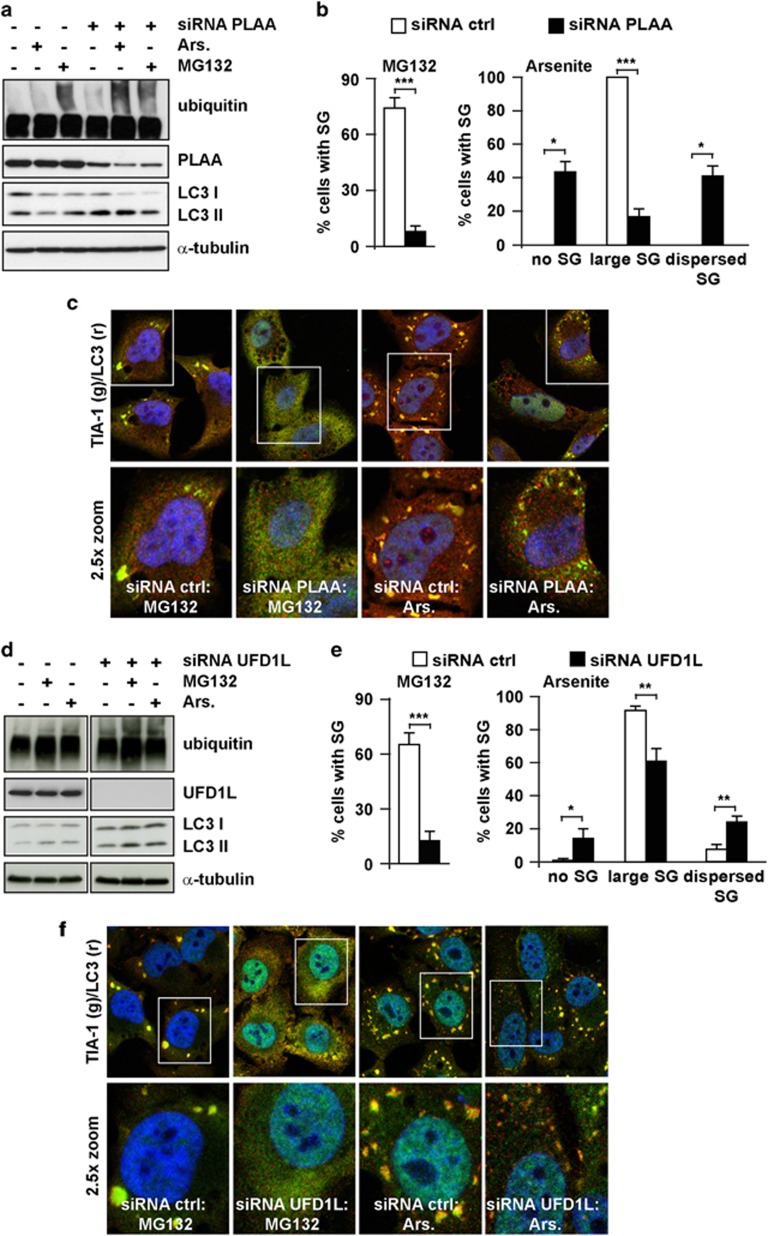
Depletion of the VCP co-factors PLAA and UFD1L affects SG formation. HeLa cells lipofected with control (ctrl), PLAA (**a–c**) or UFD1L siRNA (**d**–**f**) for 72 h were left untreated (data not shown) or treated for 3 h with MG132 or 45 min with arsenite (Ars). Cells were lysed and processed for western blot (**a**, **d**), or fixed and labeled with anti-TIA-1, LC3 and DAPI (**c**, **f**). (**b**, **e**) Quantitation of data in **c** and **f**. Error bar, S.E.M. ***P<0.001; **P<0.01; *P<0.05. (**c**, **f**) 2.5 × magnification of the selected area. See also Supplementary Figure S4

Prior to SG formation, polysomes disassemble and release ubiquitinated un/misfolded proteins, DRIPs and ribosomal subunits (Figure 3f). DRIPs, which represent ca the 30% of newly synthesized proteins,^4^ are aggregation-prone and may possibly affect the reversible self-aggregation of TIA-1, which drives SG formation.^6^ In addition, while the small ribosomal subunit (40S) is a component of SGs, the large one (60S) is excluded from and antagonistic to SGs.^22, 32^ To enable client selection, VCP interacts with co-factors, like ubiquitin fusion degradation 1 like (UFD1L) and phospholipase A2-activating protein (PLAA). UFD1L promotes with VCP the degradation of DRIPs bound to translating ribosomes^33^ (Figure 3f). PLAA forms a complex that also contains HDAC6, a SG component.^34^ This complex modulates ubiquitin turnover and client transfer to HDAC6 for targeting to aggresome.^34, 35, 36^ VCP and PLAA are also required in yeast for ribophagy, the lysosome-mediated degradation of 60S^37, 38^ (Figure 3f). We speculated that defective clearance of DRIPs and 60S mediated by lysosome/autophagy, proteasome and VCP (and co-factors), may contribute to impaired SG growth. In line with this hypothesis, knockdown of PLAA (Figures 4a–c) or UFD1L (Figures 4d–f) impaired MG132 and arsenite-induced SGs, although their effect was less severe than VCP knockdown. Silencing VCP, PLAA and UFD1L also inhibited heat shock-induced SGs (Supplementary Figure S4A). Knockdown of VCP, PLAA or UFD1L did not induce Hsp70 (Supplementary Figure S4B) or apoptosis/PARP cleavage (Supplementary Figures S4B–D; PARP was slightly cleaved in VCP-depleted cells). As positive control, PARP cleavage was induced with doxorubicin and was blocked by co-treatment with zVAD-fmk (Supplementary Figure S4C). Likewise, co-treatment with zVAD-fmk could not rescue the inhibitory effect of VCP or PLAA knockdown on SG assembly (data not shown). Finally, SG response was not affected in cells where ubiquitin-like-domain-containing protein Ubxd8 (Ubxd8), another VCP co-factor, was silenced (Supplementary Figures S4E–G). The VCP–Ubxd8 complex promotes the release of ubiquitinated HuR from ribonucleoprotein complexes;^39^ HuR is a component of SGs but it is not required for their assembly.^40^ Thus, only a subset of VCP complexes affects SGs.

### DRIPs are excluded but adjacent to SGs

**Figure 5:**
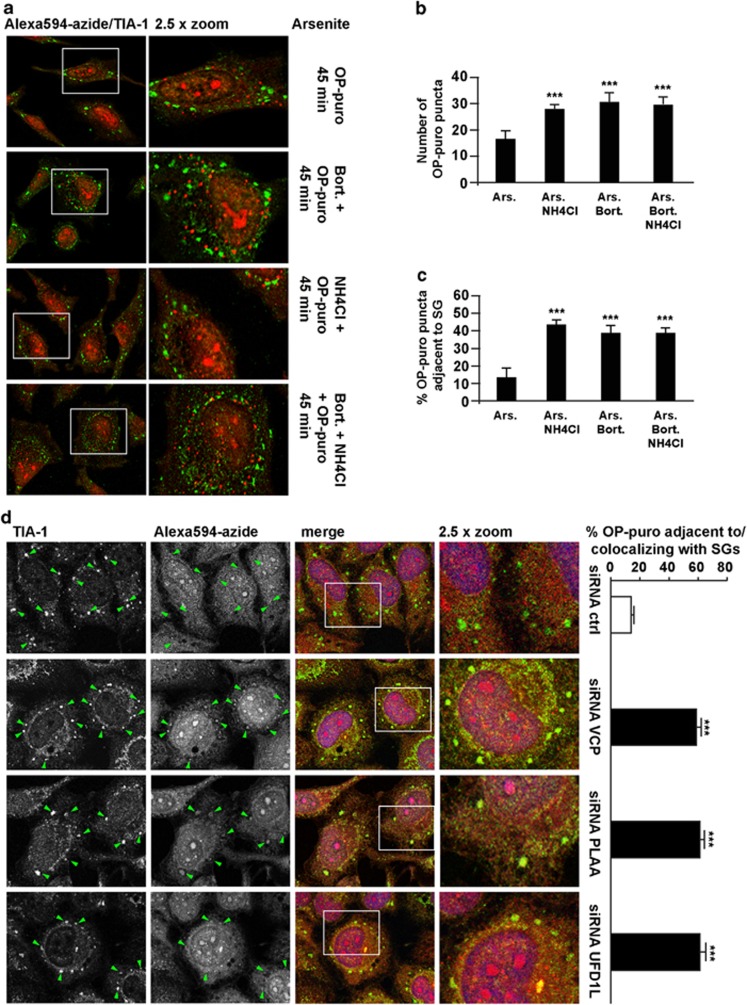
Inhibition of proteasome and lysosome or silencing of VCP and co-factors lead to the accumulation of OP-puro-labeled DRIPs adjacent to or within SGs. (**a**–**c**) HeLa cells were co-treated for 45 min with OP-puro and arsenite (Ars.); where indicated, cells were pretreated with bortezomib (Bort.) overnight and/or ammonium chloride (NH_4_Cl) for 2 h 15 min. Cells were fixed and labeled with Alexa594–Azide and anti-TIA-1. (**b**) The number of OP-puro puncta per cell is shown. Error bar, S.E.M. ***P<0.001 compared with Ars. (**c**) The percentage of OP-puro puncta adjacent to/colocalizing with SGs is shown. Error bar, S.E.M. ***P<0.001 compared with Ars. (**d**) HeLa cells lipofected for 72 h with control, VCP, PLAA or UFD1L siRNA were treated for 45 min with OP-puro and Ars., fixed and stained with Alexa594–Azide, anti-TIA-1 and DAPI. Arrowheads indicate OP-puro-labeled DRIPs colocalizing with TIA-1-positive SGs. The percentage of OP-puro puncta adjacent to/colocalizing with SGs is shown. Error bar, S.E.M. ***P<0.001 compared with cells transfected with control siRNA. (**a**, **d**) 2.5 × magnification of the selected area. See also Supplementary Figure S5

To test whether accumulation of DRIPs occurs and correlates with decreased SG formation, we labeled nascent chains using O-propargyl-puromycin (OP-puro).^25^ Forty-five minutes after treatment, aggregated OP-puro-labeled DRIPs (stained with Alexa594–Azide) accumulated in the cytosol (Supplementary Figures S5A and B). Only a background Alexa594–Azide signal was detectable after co-treatment with OP-puro and cycloheximide, a translation inhibitor, demonstrating the specificity of OP-puro (Supplementary Figure S5C). While at the concentration and time used, OP-puro did not by itself induce SGs (Supplementary Figure S5B), concomitant treatment with arsenite and OP-puro induced SGs (Supplementary Figure S5D). DRIPs were excluded from SGs, but could be found adjacent to SGs (Supplementary Figure S5D, arrowheads). These OP-puro-labeled products colocalized with ubiquitin (Supplementary Figure S5E) and the autophagy adaptor sequestosome 1 (SQSTM1; Supplementary Figure S5F), which can target certain ubiquitinated proteins, including DRIPs, to autophagosomes/lysosomes.^41^ Indeed, partial colocalization of DRIPs with LAMP2 (lysosomal associated membrane protein-2) was found after co-treatment with OP-puro and arsenite, supporting the fact that DRIPs released by polysomes can be targeted to lysosomes (Supplementary Figure S5G, arrowheads). Interestingly, while ubiquitin is a component of SGs^8^ and colocalizes with DRIPs adjacent to SGs (Supplementary Figures S5E and H), SQSTM1 only colocalized with nascent chains and was excluded from SGs (Supplementary Figures S5F and I). SQSTM1 adjacency to SGs further points to a possible link between extraction of ubiquitinated proteins, degradation systems and SG assembly. Inhibition of proteasome and/or lysosome significantly increased the number of OP-puro-labeled cytoplasmic puncta suggesting that DRIPs are targeted to proteasome and lysosome for disposal (Figures 5a and b). Bortezomib and NH_4_Cl also increased the adjacency of DRIPs to SGs (Figures 5a and c). Thus, if not properly degraded, nascent peptides accumulate in the close vicinity of assembling SGs.

VCP, PLAA or UDF1L, which promote release and degradation of nascent proteins from the ribosome^33^ and which regulate SG assembly, colocalize with OP-puro-labeled peptides (Supplementary Figures S5J–L). While arsenite-induced SGs did not contain DRIPs in control cells (Figure 5d), OP-puro-labeled peptides significantly colocalized with TIA-1 in SGs in VCP-, PLAA- or UFD1L-deficient cells (Figure 5d). Thus, inhibition of lysosomes, VCP, PLAA and UFD1L leads to formation of altered SGs that are adjacent to/contain undigested DRIPs.

### RPL19 is retained in SGs that form in cells with impaired autophagy, lysosome or VCP

**Figure 6:**
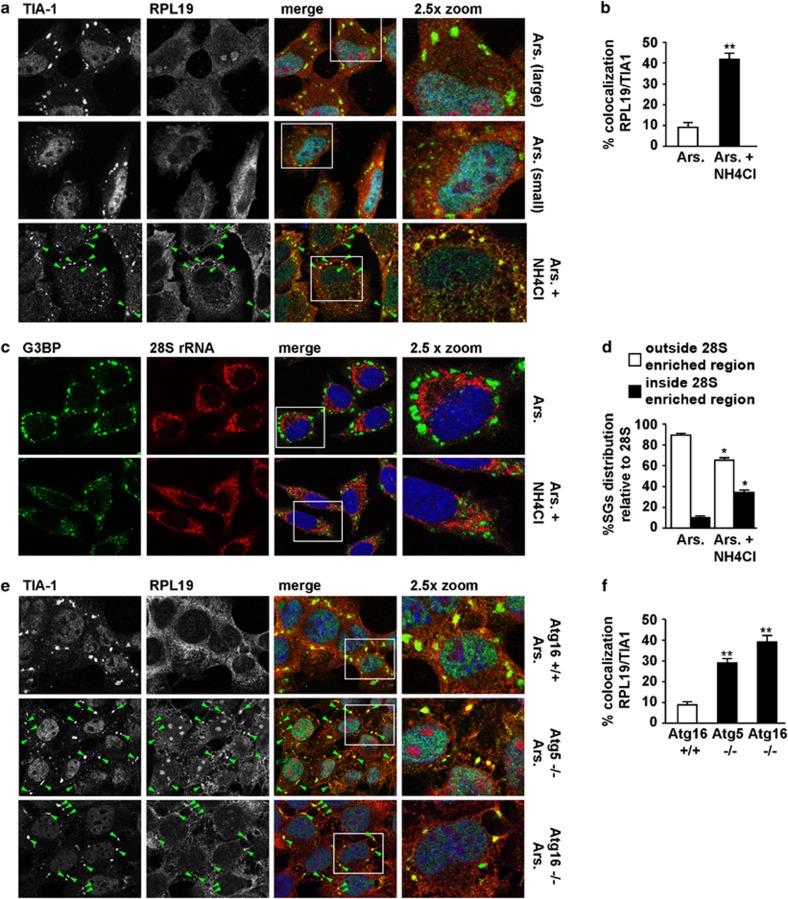
Chemical inhibition of lysosomes or genetic perturbation of autophagy leads to formation of SGs that contain RPL19. (**a**, **b**) HeLa cells were treated for 45 min with arsenite (Ars.) alone or following ammonium chloride (NH_4_Cl) pre-treatment. Cells were fixed and labeled with anti-TIA-1, RPL19 and DAPI. (**b**) Quantitation of colocalization of RPL19 and TIA-1 is shown. Error bar, S.E.M. **P<0.01. (**c**, **d**) Cells treated as described in **a** were fixed, subjected to *in situ* hybridization with a 28S rRNA probe coupled to Alexa594 and labeled with anti-G3BP and DAPI. (**d**) Quantitation of the % of TIA-1-positive SGs located outside or inside the 28S rRNA perinuclear-enriched region is shown. Error bar, S.E.M. *P<0.05. (**e**, **f**) Atg16^+/+^, Atg5^−/−^ and Atg16^−/−^ MEFs were treated for 45 min with Ars. and labeled with anti-TIA-1, RPL19 and DAPI. (**f**) Quantitation of colocalization of RPL19 and TIA-1 is shown. Error bar, S.E.M. **P<0.01. (**a**–**c**) 2.5 × magnification of the selected area. See also Supplementary Figure S6

**Figure 7:**
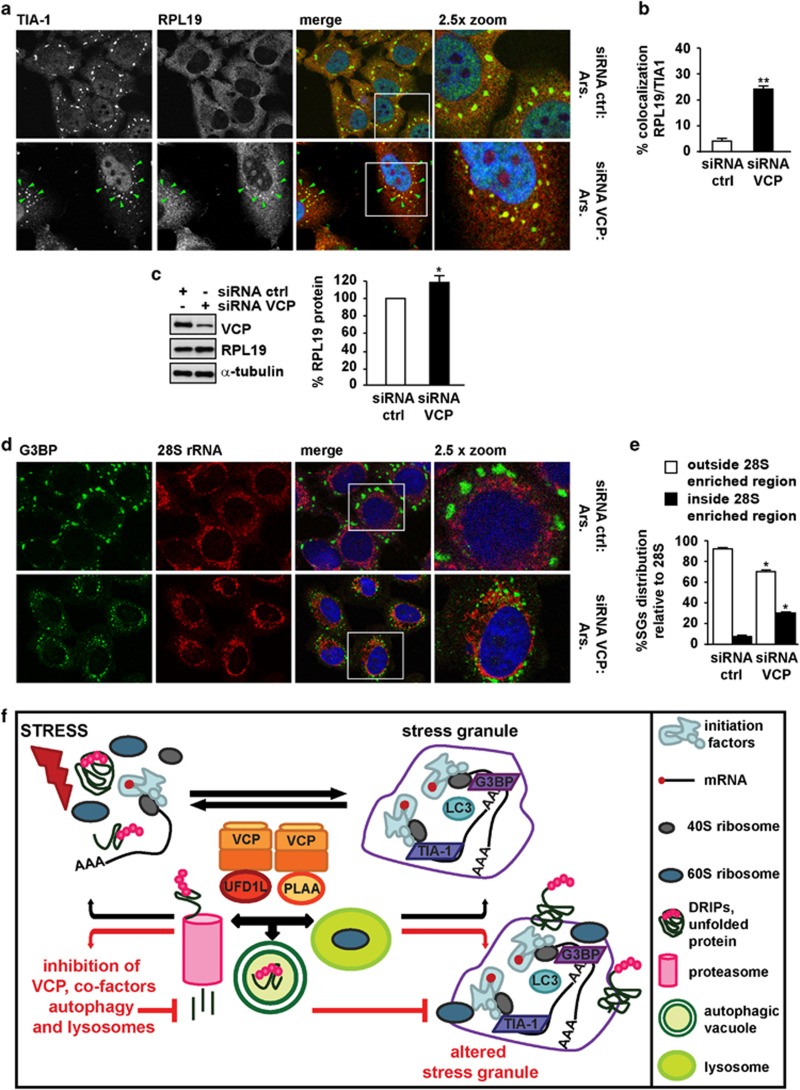
RPL19 and 28S rRNA partly colocalize with arsenite-induced SGs in VCP-depleted cells. HeLa cells lipofected for 72 h with control or VCP siRNA were treated for 45 min with arsenite (Ars.), fixed and labeled with anti-TIA-1, RPL19 and DAPI (**a**, **b**) or subjected to western blot (**c**) or to *in situ* hybridization with a 28S rRNA probe, followed by co-staining with anti-G3BP and DAPI (**d**, **e**). (**b**) Quantitation of colocalization of RPL19 and TIA-1 is shown. Error bar, S.E.M. **P<0.01. (**c**) Quantitation of RPL19 protein levels is shown. Error bar, S.E.M. *P<0.05. (**e**) Quantitation of the % of TIA-1-positive SGs located outside or inside the 28S rRNA perinuclear-enriched region is shown. Error bar, S.E.M. *P<0.05. (**f**) Schematic model of the proposed interplay between VCP, autophagy and SGs. Upon stress polysomes disassemble releasing DRIPs and ribosome subunits (40S and 60S), while mRNA-binding proteins containing prion-like domains (e.g., TIA-1) trigger the sequestration of bound mRNAs into SGs. SGs also contain, besides initiation factors, 40S and the autophagy protein LC3. Instead, DRIPs and 60S are excluded from assembled SGs. DRIPs and ubiquitinated unfolded proteins are targeted to degradation by proteasome and autolysosomes with the assistance of VCP and its co-factors UFD1L and PLAA. Also damaged 60S can be targeted to degradation by proteasome and/or lysosome, a process that also involves VCP and co-factors, while undamaged 60S can be recycled (not shown). Inhibition of autophagy or lysosomes, as well as depletion of VCP (and the co-factors PLAA or UFD1L) leads to the accumulation of DRIPs and 60S within the foci where SGs are assembling. Under such conditions, the SG response is decreased (in term of SG size and number) and SGs contain the non-canonical components DRIPs and 60S. (**a**, **d**) 2.5 × magnification of the selected area

Upon proteotoxic stress, polysomes disassemble and also release, besides DRIPs, 40S and 60S ribosomes. While 40S are recruited into SGs (Supplementary Figure S6A), 60S are excluded from SGs and antagonize SG formation^22, 32^ (Figure 6a). Nonfunctional 60S degradation is regulated by diverse processes, including VCP–UFD1 dissociation,^42^ which occurs following proteasome inhibition and treatment with arsenite;^42, 43^ ribophagy,^37^ which requires four players that also modulate SG assembly: VCP (Cdc48), PLAA (Ufd3), USP10 (Ubp3) and G3BP (Bre5)^13, 14, 37, 38^ (and our results; Figures 3 and 4); and lysosomes, which directly digest rRNA.^44^ Thus, we asked whether 60S accumulate adjacent to/within assembling SGs after autophagy, lysosome or VCP inhibition, analogous to what we observed with DRIPs. We assessed ribosomal protein L19 (RPL19) and 28S rRNA, both 60S components. RPL19 was devoid from both large and smaller SGs that accumulated in HeLa cells treated with arsenite, while it significantly colocalized with TIA-1 in SGs following co-treatment with arsenite and NH_4_Cl (Figures 6a and b). In line with this, 28S rRNA was excluded from the large SGs induced by arsenite (Figure 6c), but showed a perinuclear-enriched distribution with the SGs generally aligned outside this region. Following exposure to arsenite and NH_4_Cl, we observed a more disorganized distribution of SGs that were smaller in size and accumulated in the 28S rRNA perinuclear-enriched region (Figures 6c and d). Similar to HeLa cells, arsenite-treated autophagy-proficient cells formed SGs that did not contain RPL19 (Figures 6e and f). Instead, RPL19 and TIA-1 colocalized in arsenite-induced SGs in Atg5^−/−^ and Atg16^−/−^ cells (Figures 6e and f). In autophagy-proficient cells co-treated with arsenite and NH_4_Cl, TIA-1 colocalization with RPL19 was similar to that seen in autophagy-deficient cells (Supplementary Figure S6C). Finally, VCP-depleted cells showed increased colocalization of RPL19 with TIA-1-positive SGs (Figures 7a and b) and displayed higher basal levels of RPL19 (Figure 7c). The spatial distribution of SGs and 28S rRNA was also affected in VCP-deficient cells, where SGs accumulated in the 28S rRNA-enriched perinuclear region (Figures 7d and e). Together, our results demonstrate that cells with inhibited autophagy, lysosome or VCP form SGs with altered morphology that retain 60S components within or in their close vicinity.

## Discussion

The finding that SGs are targeted to autophagy for degradation by VCP^15^ and that SG disassembly requires chaperone-driven protein disaggregation^45^ are consistent with the idea that PQC may survey and/or assist SG dynamics, both assembly and disassembly. However, despite these observations, it was unclear whether the PQC actively participates in SG assembly and whether impairments thereof affect SGs.

Our data demonstrate that inhibition of autophagy, lysosomes and VCP impairs SGs, supporting that the PQC modulates SG formation. Autophagy, lysosomes and VCP govern protein (and organelle) degradation. In cells with impaired autophagy, lysosome or VCP function, SGs were smaller in size as compared with SGs forming in control cells. This suggests that specific components may need to be extracted from the foci where SGs assemble to be targeted to degradation; this may indirectly contribute to optimal SG growth. DRIPs and 60S are released by disassembling polysomes, prior to SG assembly, and are among the clients that are cleared with the assistance of autophagy, lysosomes and VCP.^3, 4, 18, 22, 33, 46, 47, 48^ We found that DRIPs are excluded from arsenite-induced SGs; however, upon UPS and lysosome inhibition, DRIPs accumulated adjacent to SGs. DRIPs also significantly colocalized with SGs in VCP-, UFD1- and PLAA-depleted cells, consistent with their role in handling and degradation of DRIPs. These results suggest that impaired extraction of DRIPs from the foci where SGs assemble may affect SG composition and morphology (Figure 7f).

Ribosomes are also released by disassembling polysomes. Only the 40S is a component of SGs, while the 60S is excluded and impedes SG assembly.^22^ 28S rRNA is enriched in the perinuclear region and SGs form in the close vicinity of, but always outside, this region. Instead, upon autophagy, VCP or lysosomal inhibition, SGs tend to accumulate within the perinuclear 28S rRNA-enriched region. Moreover, RPL19 colocalized with SGs in cells with impaired autophagy, lysosome or VCP. Consistent with their role in ribophagy and SG assembly^13, 14, 38^ (and Figures 3 and 4), inhibition of VCP, autophagy or lysosome alters SG composition, morphology and 60S distribution. This suggests that VCP may participate in extracting 60S from the foci where SGs are assembling. 60S can be next recycled or, when damaged due to the proteotoxic stress that elicits SGs, targeted to degradation via ribophagy, or both (Figure 7f). To what extent 60S are degraded or recycled, allowing polysome re-assembly, is currently unknown. Our findings that RPL19 was trapped within SGs in cells with inhibited autophagy/lysosome and VCP suggest that autolysosome-based degradation of some 60S occurs concomitantly to SG assembly. At this stage we cannot exclude that, besides DRIPs and 60S, other components accumulate within SGs with altered morphology that form in cells with proteostasis (autophagy, lysosome and VCP) dysfunction, and that such additional components may be major causes of the impaired SG assembly. An important challenge for future work will be to identify how VCP and co-factors orchestrate the selection of components to be extracted from SGs and understanding LC3 role in SGs.

The discovery that autophagy, lysosome and VCP inhibition affects SG morphology and composition implies that proteostasis imbalances will have a direct impact on SGs. This may render the cells vulnerable under challenging/disease conditions. Indeed, recent data implicate SGs and deregulated proteostasis in amyotrophic lateral sclerosis, frontotemporal lobar degeneration and multisystem proteinopathy, which are also associated with VCP mutations^49, 50, 51, 52, 53, 54^ and where protein aggregates that contain SG components accumulate. Thus, inappropriate SG dynamics may be relevant to pathogenesis. Our data are in line with this hypothesis and demonstrate that SGs forming under conditions of autophagy/lysosome or VCP inhibition accumulate non-canonical components (DRIPs and 60S); this in turn, can impair SG dynamics and contribute to SG persistency. Persistent or partly disassembled SGs, if not properly disposed,^15^ may act as seeds for aggregation, further challenging protein and RNA homeostasis. In parallel, SGs sequester pro-apoptotic factors, indirectly inhibiting apoptosis.^10, 13, 55^ Although we could not detect any major sign of toxicity under our experimental conditions (in line with the findings from Buchan *et al.*^15^), we cannot exclude that, as a consequence of proteostasis imbalances, chronic-impaired SG formation may lead to, for example, deregulation of signaling pathways and decreased sequestration of pro-apoptotic factors, which could also contribute to cell vulnerability/death.

## Materials and Methods

### Cell culture, treatments and transfection

HeLa cells, Atg16^+/+^, Atg16^−/−^, Atg5^+/+^, Atg5^−/−^and m5-7 MEFs were cultured in DMEM (ECB7501L; EuroClone, Milan, Italy) supplemented with 2 mM L-glutamine, 100 U/ml penicillin/streptomycin and 10% fetal bovine serum (Lonza, Basel, Switzerland) in a 37 °C incubator with 5% CO_2_.

Atg5^+/+^ and Atg5^−/−^ MEFs were kindly provided by Dr. T Yoshimori (Osaka University). Transfections were performed using Lipofectamine 2000 (Life Technologies, Monza, Italy) according to the manufacturer's instructions. All siRNAs used were from Dharmacon/GE Healthcare (Milan, Italy): siGENOME non-targeting control siRNA, ON-TARGETplus VCP siRNA, ON-TARGETplus PLAA siRNA, ON-TARGETplus UFD1L siRNA and ON-TARGETplus Ubxd8 siRNA.

Cells were treated with the following drugs at the concentrations indicated here: arsenite 0.5 mM or, when specified, 0.1 mM; Z-Leu-Leu-Leu-al (MG132) 20 *μ*M; ammonium chloride (NH_4_Cl) 20 mM; CLQ 50 *μ*M; ML240 5 *μ*M; EerI 10 *μ*M; Bortezomib (Bort.) 100 nM; OP-puro 25 *μ*M.

### Immunofluorescence microscopy

Unless otherwise indicated, cells were grown on coverslip, treated as indicated, washed with cold PBS and fixed with 3.7% formaldehyde in PBS for 9 min at room temperature, followed by permeabilization with cold acetone for 5 min at −20 °C. Blocking and incubation with primary and secondary antibodies were performed in PBS containing 3% BSA and 0.1% Triton X-100. Primary and secondary antibodies used are listed below. Analysis of the cells was done by confocal imaging using a Leica SP2 AOBS system (Leica Microsystems, Milan, Italy) and a 63 × oil-immersion lens.

### OP-puro labeling of cultured cells

The protocol for OP-puro labeling was adapted from Liu *et al.*^25^ Cells were incubated with 25 *μ*M OP-puro for 45 min and CuAAC detection of OP-puro incorporated into nascent proteins was performed as previously described, using Alexa Fluor 594 azide (A10270, Life Technologies) (Liu J *et al.*^25^). Cells were next processed for immunofluorescence microscopy as described above.

### *In situ* hybridization

The *in situ* hybridization protocol was adapted from Reineke *et al.*^56^ Cells were fixed with 2% formaldehyde in PBS for 1 min at room temperature and permeabilized with ice-cold methanol for 10 min at −20 °C. The following 5′-biotinylated DNA probe was used to detect 28S rRNA: 5′-cggcgctgccgtatcgttccgcctgggc gggattctgacttagaggcgttc-3′. The probe was hybridized in hybridization buffer (50% formamide, 2 × SSC, 10% dextran sulfate, 0.2% BSA, 5 mM DTT) at a final concentration of 1 *μ*g/ml for 24 h at 43 °C in a humidified chamber and detected by subsequent incubation with Cy3 streptavidin (GE Healthcare Amersham, Milan, Italy) in 4 × SSC plus 0.1% Triton X-100 for 1 h at room temperature. Blocking and incubation with primary and secondary antibodies were next performed as described above.

### Western blotting

Cells were lysed in Laemmli buffer and protein samples were boiled 3 min at 100 °C, separated by SDS-PAGE, transferred onto nitrocellulose membranes, subjected to western blot analysis, and visualized using an ECL detection kit (Thermo Scientific, Milan, Italy).

### Antibodies and reagents

The primary antibodies used are listed. Mouse anti-LAMP2 (H4B4), mouse anti-PARP-1(F-2), mouse anti-PLAA (E-1), mouse anti-Ribosomal Protein L19 (K-12), mouse anti-SQSTM1 (D-3), mouse anti-UFD1 (19), rabbit anti-SQSTM1 (H-290), rabbit anti-ETEA (H-300) and goat anti-TIA-1(C-20) were from Santa Cruz Biotechnology Inc. (Heidelberg, Germany). Mouse anti-G3BP and mouse anti-HSP70 (SMC-100A/B) were from BD Biosciences (Milan, Italy) and StressMarq Biosciences Inc., (Victoria, BC, Canada) respectively. Mouse anti-Ribosomal Protein L19 and mouse anti-VCP were from Abnova (Tapei, Taiwan) and Thermo Scientific, respectively. Mouse anti-*α*-tubulin and rabbit anti-phospho eIF2α were from Sigma-Aldrich (Milan, Italy). Rabbit anti-LC3 (Novus Biologicals Ltd, Cambridge, UK) was used in the majority of our experiments, unless otherwise indicated.

The secondary antibodies used are listed. All the Alexa-conjugated secondary antibodies were from Life Technologies: donkey-anti-goat-Alexa488, donkey-anti-mouse-Alexa594, donkey-anti-rabbit-Alexa594, donkey-anti-rabbit-Alexa488 and donkey-anti-mouse-Alexa488. Mouse and rabbit HRP-conjugated secondary antibodies for western blot were from GE Healthcare Europe GmbH (Milan, Italy).

The reagents used in this study are as follows: ammonium chloride (A9434), chloroquine (C6628), OP-puro (P8833), sodium arsenite (Carlo Erba Reagents, Cornaredo, Italy), z-Leu-Leu-Leu-al (MG132; C2211), cycloheximide (C7698) and tetracycline hydrochloride (T7660) were from Sigma-Aldrich. EerI (sc-358130) was from Santa Cruz Biotechnology, Inc. Bort. (S1013) was from Selleck Chemicals (Munich, Germany); ML240 was a kind gift from Prof. RJ Deshaies.

### Statistics, analysis of SG size and quantification of colocalization

Student's *t*-test was used for comparisons between two groups. One-way ANOVA followed by Bonferroni–Holm *post-hoc* test was used for comparisons between three or more groups. *P<0.05; **P<0.01; ***P<0.001.

SG size was measured using ImageJ software (http://rsb.info.nih.gov/ij/). Average size of 300 SGs is reported.

Colocalization efficiency of RPL19 with TIA-1 in SGs was performed using ImageJ software (Image J, Colocalization Coloc 2, Manders' correlation). Region-of-Interest (ROI) were drawn around single cells. Background and threshold correction were applied for each ROI.

## Abbreviations

ATG: autophagy gene
DRIPs: defective ribosomal products
eIF2α: alpha subunit of translation initiation factor 2
G3BP: Ras-GTPase-activating protein SH3 domain-binding protein
HDAC6: histone deacetylase 6
Hsp70: 70 kd heat shock protein
LAMP2: lysosomal associated membrane protein-2
PARP: poly ADP ribose polymerase
PLAA: phospholipase A2-activating protein
PQC: protein quality control
RPL19: ribosomal protein L19
RPS6: ribosomal protein S6
SG: stress granules
SQSTM1: sequestosome 1
TIA-1: T-cell-restricted intracellular antigen-1
Ubxd8: ubiquitin-like-domain-containing protein Ubxd8
UFD1L: ubiquitin fusion degradation 1 like
UPS: ubiquitin–proteasome system
USP10: ubiquitin specific peptidase 10
VCP: valosin-containing protein
EerI: eeyarestatin I
OP-puro: O-propargyl-puromycin
Bort.: bortezomib
ROI: region-of-Interest
CLQ: chloroquine
