# Supplementary material for: Inhibition of autophagy, lysosome and VCP function impairs stress granule assembly
Source: Cell Death Differ. 2014 Jul 18;21(12):1838–51. doi: 10.1038/cdd.2014.103 (PMC4227144; doi:10.1038/cdd.2014.103)
Supplement: Supplementary Material [file cdd2014103x1.doc]

**SUPPLEMENTAL FIGURE LEGENDS**

**Figure S1. Lysosomal inhibitors impair rather than delaying SG formation, Related to Figure 1.**

(A) HeLa cells were treated with 20 µM MG132 alone or with 20 mM NH_4_Cl for 1 to 8 hr and either processed for ubiquitin, LC3 I, LC3 II and α-tubulin western blotting. Scatter plot shows percentage of cells with TIA-1 positive SGs at different time points.

(B, C) HeLa cells were left untreated (ctrl) or treated with 20 µM MG132 and/or 20 mM NH_4_Cl for 3 hr and extracted in polysome buffer (T: total extract). Nuclei and debris (P1) were removed by microfuge centrifugation. Postnuclear supernatants (S1) were layered onto a 45% sucrose cushion for concentration, resulting in a supernatant fraction (S2) and a pelleted polysome fraction. (B) Expression levels of ubiquitin, LC3 I, LC3 II and RPS6 were analyzed in the following fractions: T, S1, P1 and S2. # likely corresponds to LC3 T. (C) Pelleted polysomes were resuspended in polysome buffer; polysomes from control cells were divided into two fractions that were either left untreated (control w/o EDTA) or treated with 40 mM EDTA to dissociate polysomes (+EDTA). Polysomes were then separated on a linear 15-45% sucrose gradient. Individual fractions from the sucrose gradients were subjected to western blot analysis to localize RPS6. The lightest fractions are at the left. Non-specific reactive bands are marked (*). Pellet corresponds to the bottom of the gradients; proteins were directly solubilized in Laemmli buffer. Addition of EDTA, used as positive control, caused a shift of RPS6 towards the low molecular weight fractions (Figure S1C; lane 3 and 4).

(D) HeLa cells were left untreated (-) or pretreated with 20 mM NH_4_Cl for 2 hr 15 min prior to addition of 0.5 mM Sodium Arsenite (Ars.) for 45 min. 10 µg/ml Puromycin was added during the last 10 min of treatment to monitor translation. Cells were processed for puromycin, ubiquitin, LC3 and α-tubulin western blot.

**Figure S2. LC3 is recruited into SGs.**

(A) Hela cells left untreated (ctrl) or pretreated with 20 mM NH_4_Cl for 2 hr 15 min prior to addition of 0.5 mM Sodium Arsenite (Ars.) for 45 min. Cells were fixed and labeled for total RNA using SYTO RNASelect, followed by labeling with anti-TIA-1 and DAPI. Low (l.e.) and high (h.e.) exposure times are shown. Arrowheads indicate RNA-positive SGs.

(B) HeLa cells were treated overnight with 200 µM Leupeptin (L), 10 µg/mL E64d together (E) and/or 10 µg/mL Pepstatin A (P). Cells were next treated with 20 µM MG132 for 3 hr and either processed for immunofluorescence, using anti-TIA-1, anti-LC3 and DAPI or for LC3 and α-tubulin western blot. Histogram shows percentage of cells with TIA-1 positive SGs. Error bar, SEM. one way ANOVA followed by Bonferroni-Holm post-hoc test: **p<0.01; *p<0.05 compared to MG132 condition.

(C) HeLa cells were treated for 45 min with 0.5 mM Sodium Arsenite, fixed and labeled with anti-TIA-1 and two different anti-LC3 antibodies (anti-LC3-L7543 or anti-LC3#11) and DAPI. Arrowheads indicate LC3-positive SGs.

(D) Atg5^+/+^ and Atg5^-/-^ MEFs were treated for 45 min with 0.5 mM Sodium Arsenite, fixed and labeled with anti-TIA-1, anti-LC3 (Novus Biologicals) and DAPI. Arrowheads indicate LC3-positive SGs.

(A, B, C, D) 2.5x magnification of the selected area.

**Figure S3. Chemical inhibition of proteasome and/or lysosome affects SG assembly and clearance, Related to Figure 1.**

(A) HeLa cells were treated for 45 min with 0.5 mM Sodium Arsenite (Ars.), washed 3 times with growth medium (washout) and allowed to recover for 1 or 2 hr in fresh medium alone (+ rec.) or supplemented with either 20 mM NH_4_Cl (+ NH_4_Cl) or 50 µM Chloroquine (+ CLQ). Cells were fixed and labeled with anti-TIA-1 coupled to a green (g) secondary antibody and anti-G3BP coupled to a red (r) secondary antibody. Percentage of cells containing large (L) or small and dispersed (S) SGs are shown.

(B, C) HeLa cells were pretreated for 2 hr 15 min with 20 µM MG132 alone or in combination with 20 mM NH_4_Cl, followed by incubation with 0.5 mM Sodium Arsenite (Ars.) for 45 min. Cells were fixed and labeled with anti-TIA-1, anti-LC3 and DAPI. (C) Histogram shows percentage of cells with TIA-1 positive large SGs, dispersed SGs and no SGs. Error bar, SEM. one way ANOVA followed by Bonferroni-Holm post-hoc test: ***p<0.001.

(D, E) HeLa cells were left untreated or pretreated overnight with 100 nM Bortezomib (Bort.) ^1^, followed by exposure to 0.5 mM Sodium Arsenite (Ars.) for 45 min. Cells were fixed and labeled with anti-TIA-1, anti-LC3 and DAPI. (E) Histogram shows percentage of cells with TIA-1 positive large SGs, dispersed SGs and no SGs. Error bar, SEM. one way ANOVA followed by Bonferroni-Holm post-hoc test: ***p<0.001; **p<0.01.

(F) HeLa cells were left untreated or treated with either 20 µM MG132 and/or NH_4_Cl 20 mM for 3 hr; where indicated, cells were incubated with 0.5 mM Sodium Arsenite for 45 min. Cells were processed for ubiquitin, inducible HSP70, LC3 I, LC3 II and α-tubulin western blot.

(G) HeLa cells were left untreated or treated overnight with 100 nM Bortezomib (Bort.), 200 µM Leupeptin (L), 10 µg/mL E64d (E) and/or 10 µg/mL Pepstatin A (P). Cells were processed for western blot as described in F.

(H) HeLa cells were treated as described in F and processed for ubiquitin, phopsho-eIF2α and α-tubulin western blot.

(I) HeLa cells were treated with 20 µM MG132 in combination with 25 µM z-VAD-fmk and NH_4_Cl 20 mM for 3 hr; where indicated, cells were incubated with 0.5 mM Sodium Arsenite (Ars.) for 45 min, prior to fixation. The cells were fixed and labeled with anti-TIA-1. The percentage of cells with TIA-1 positive SGs for MG132 treatments, and with TIA-1 positive large (L) or small and dispersed (S) SGs were counted, respectively.

(B, D) 2.5x magnification of the selected area.

**Figure S4. Depletion of VCP, PLAA and UFD1L, but not of Ubxd8, impairs SG formation, Related to Figures 3 and 4.**

(A) HeLa cells lipofected with control (ctrl), VCP, PLAA or UFD1L siRNA for 72 hr were heated at 43.5°C (HS) for 45 min. Cells were fixed and labeled with anti-TIA-1 (coupled to a green (g) secondary antibody), anti-LC3 (coupled to a red (r) secondary antibody) and DAPI. The percentage of cells containing large (L) or small and dispersed (S) SGs are indicated.

(B) Cells were lipofected as described in A and total protein extracts were prepared 72 hr post-transfection. Expression levels of VCP, PLAA, UFD1L, inducible HSP70, PARP (total and cleaved-fragment; c.f.) and α-tubulin were analyzed by western blot.

(C) HeLa cells were treated with 5 µM Doxorubicine for 24 hr in presence or absence of 25 µM z-VAD-fmk. Expression levels of PARP (total and cleaved-fragment; c.f.) and α-tubulin were analyzed by western blot.

(D) Cells were lipofected as described in A and total protein extracts were prepared 72 hr post-transfection. Samples were processed for PARP (total and cleaved-fragment; c.f.), VCP, PLAA and α-tubulin western blot. Histogram shows the percentage of cleaved PARP in control and VCP-depleted cells as compared to cells treated with doxycycline (Doxo.).

(E) HeLa cells were lipofected with control (ctrl), UFD1L or Ubxd8 siRNA; 72 hr post-transfection, cells were lysed and processed for UFD1L, Ubxd8 and α-tubulin western blot.

(F, G) Cells were lipofected with control (ctrl) or Ubxd8 siRNA for 72h; cells were left untreated (data not shown), treated for 45 min with 0.5 mM Sodium Arsenite (F; Ars.) or heated at 43.5°C for 45 min (G; heat shock-HS). Cells were fixed and labeled with anti-TIA-1, anti-G3BP (F) or anti-LC3 (G) and DAPI. The percentages of cells containing large (L) or small and dispersed (S) SGs are reported.

(A, E-G) 2.5x magnification of the selected area.

**Figure S5. OP-puro labeled DRIPs are excluded from SGs but colocalize with ubiquitin and the autophagy linker SQSTM1, Related to Figure 5.**

(A-D) HeLa cells were left untreated or treated for 45 min with 25 µM OP-puro; where indicated cells were cotreated with 25 µg/mL cycloheximide (CHX) or 0.5 mM Sodium Arsenite (Ars.). Cells were fixed with cold methanol and CuAAC reaction was performed, followed by labeling with Alexa594-Azide and subsequently with anti-TIA-1 and DAPI.

(E-G) HeLa cells were incubated for 45 min with 25 µM OP-puro and 0.5 mM Sodium Arsenite (Ars.). Colocalization of OP-puro labeled DRIPs (Alexa594-Azide) with ubiquitin (E), SQSTM1 (F) and LAMP2 (G) was investigated.

(H, I) HeLa cells were treated for 45 min with 0.5 mM Sodium Arsenite (Ars.), fixed and labeled with anti-TIA-1, DAPI and either anti-ubiquitin (H) or anti-SQSTM1 (I).

(J-L) HeLa cells were treated for 45 min with 25 µM OP-puro. After fixation, OP-puro labeled DRIPs were detected as previously described, followed by staining with anti-VCP (J), anti-PLAA (K) or anti-UFD1L (L).

(A-L) 2.5x magnification of the selected area.

**Figure S6. While RPS6 is a component of SGs, RPL19 colocalizes with SGs only in cells with impaired autophagy and lysosome function, Related to Figure 6.**

(A) HeLa cells were left untreated or treated for 3 hr with 20 µM MG132 or for 45 min with 0.5 mM Sodium Arsenite (Ars.), fixed and labeled with anti-TIA-1, anti-RPS6 and DAPI.

(B, C) Atg16^+/+^, Atg5^-/-^ and Atg16^-/-^ MEFs were left untreated (ctrl; B) or treated for 2 hr 15 min with 20 mM NH_4_Cl (+NH_4_Cl) followed by addition for 45 min of 0.5 mM Sodium Arsenite (Ars.; C). Cells were then fixed and labeled with anti-TIA-1, anti-RPL19 and DAPI. (C) Quantitation of colocalization of RPL19 and TIA-1 is shown.

(A-C) 2.5x magnification of the selected area.

**SUPPLEMENTAL MATERIALS AND METHODS**

**Antibodies and Reagents**

The primary antibodies used are listed. Rabbit anti-phospho eIF2α and rabbit anti- LC3 (L7543) were from Sigma-Aldrich. The home-made rabbit anti-LC3 (serum #11) was previously described (Carra et al., JBC 2008). Mouse anti-Ribosomal Protein S6 (C-8) was from Santa Cruz Biotechnology Inc. Mouse anti-puromycin was from Millipore (clone 12D10).

The reagents used in this study are as follows: Doxorubicin Hydrochloride (D1515), Valinomycin (V0627), Pepstatin A (P5318), E-64d (E8640) and Leupeptin (L5793) were from Sigma-Aldrich; Z-VAD-FMK (sc-3067) was from Santa Cruz Biotechnology, Inc.

ON-TARGETplus Ubxd8 siRNA was from Dharmacon/Thermo Scientific.

**Labeling of RNA with SYTO RNASelect**

Cells were fixed with ice-cold methanol for 10 min at -20°C and incubated for 20 min at room temperature in PBS with 500 nM SYTO RNASelect green fluorescent cell stain (S-32703, Life Technologies). Cells were washed in PBS and subsequently processed for immunofluorescence technique. Blocking and incubation with primary and secondary antibodies were next performed as previously described.

**Labeling of nascent peptides with puromycin.**

Cells were treated with 10 µg/ml puromycin for 10 min at 37°C. Where indicated cells were pre-treated with ammonium chloride and/or arsenite, prior to addition of puromycin. Cells were lysed in Laemmli buffer and subsequently processed for western blotting.

**Sucrose Gradient analysis of polysomes**

Cells were left untreated or treated as described in the Figure legend; cells were then washed with cold PBS containing 50 µg/mL cycloheximide and extracted in polysome buffer (1% NP-40; 20 mM Tris-HCl, pH 7.4; 150 mM NaCl; 1.25 mM MgCl2; 1 mM DTT; 5 U/mL RNAse inhibitor (Euroclone); 50 µg/mL cycloheximide; Antiprotease cocktail EDTA-free (Roche)). Nuclei and debris were pelleted by centrifugation at 14,000 rpm for 15 min. The postnuclear supernatants were layered onto a 45% (wt/wt) sucrose cushion (containing also 20 mM Tris-HCl, pH 7.4; 150 mM NaCl; 1.25 mM MgCl2) and centrifuged for 2 hr at 34,000 rpm in a Beckman Coulter SW 40 Ti rotor, Swinging Bucket. Pelleted polysomes were resuspended in polysome buffer; where indicated 40 mM EDTA was added. Resuspended polysomes were layered on a linear 15-45% (wt/wt) sucrose gradient and separated for 2 hr at 34,000 rpm. Equal volume fractions were collected from the top of the gradient. Individual fractions were ethanol-precipitated to remove sucrose and to concentrate the proteins. Proteins were resuspended in reducing SDS sample buffer and processed for western blot.

**Analysis of apoptosis and mitochondrial membrane potential by fluorescence-activated cell sorting (FACS)**

Annexin V Apoptosis Detection Kit (sc-4252 AK; Santa Cruz Biotechnology, Inc) with Annexin V FITC and Propidium Iodide was used to measure apoptosis and cell death. Cells were processed as described in manufacturer’s protocol and analyzed by fluorescence-activated cell sorting (FACS). To measure mitochondrial membrane potential (MMP), cells were stained with JC-1 (Invitrogen) and subjected to FACS as previously described ^2^. As positive control to induce mitochondria depolarization, cells were treated for 10 min with 0.1 mg/ml valinomycin prior to staining with JC-1. Samples were analyzed using a 16-parameter CyFlow ML flow cytometer (Partec GmbH, Munster, Germany). Data were acquired in list mode by using FloMax (Partec) software and then analyzed by Flow Jo 9.7.5 (TreeStar Inc., Ashland, Oregon, USA) under Mac OS 10. A minimum of 1 x 10^5^ cells per sample were acquired.

**SUPPLEMENTAL REFERENCES**

1. Fournier MJ, Gareau C, Mazroui R. The chemotherapeutic agent bortezomib induces the formation of stress granules. Cancer Cell Int 2010, 10: 12.

2. Troiano L, Ferraresi R, Lugli E, Nemes E, Roat E, Nasi M, et al. Multiparametric analysis of cells with different mitochondrial membrane potential during apoptosis by polychromatic flow cytometry. Nature protocols 2007, 2(11): 2719-2727.
