# Supplementary figures and images for: Inhibition of autophagy, lysosome and VCP function impairs stress granule assembly
Source: Cell Death Differ. 2014 Jul 18;21(12):1838–51. doi: 10.1038/cdd.2014.103 (PMC4227144; doi:10.1038/cdd.2014.103)

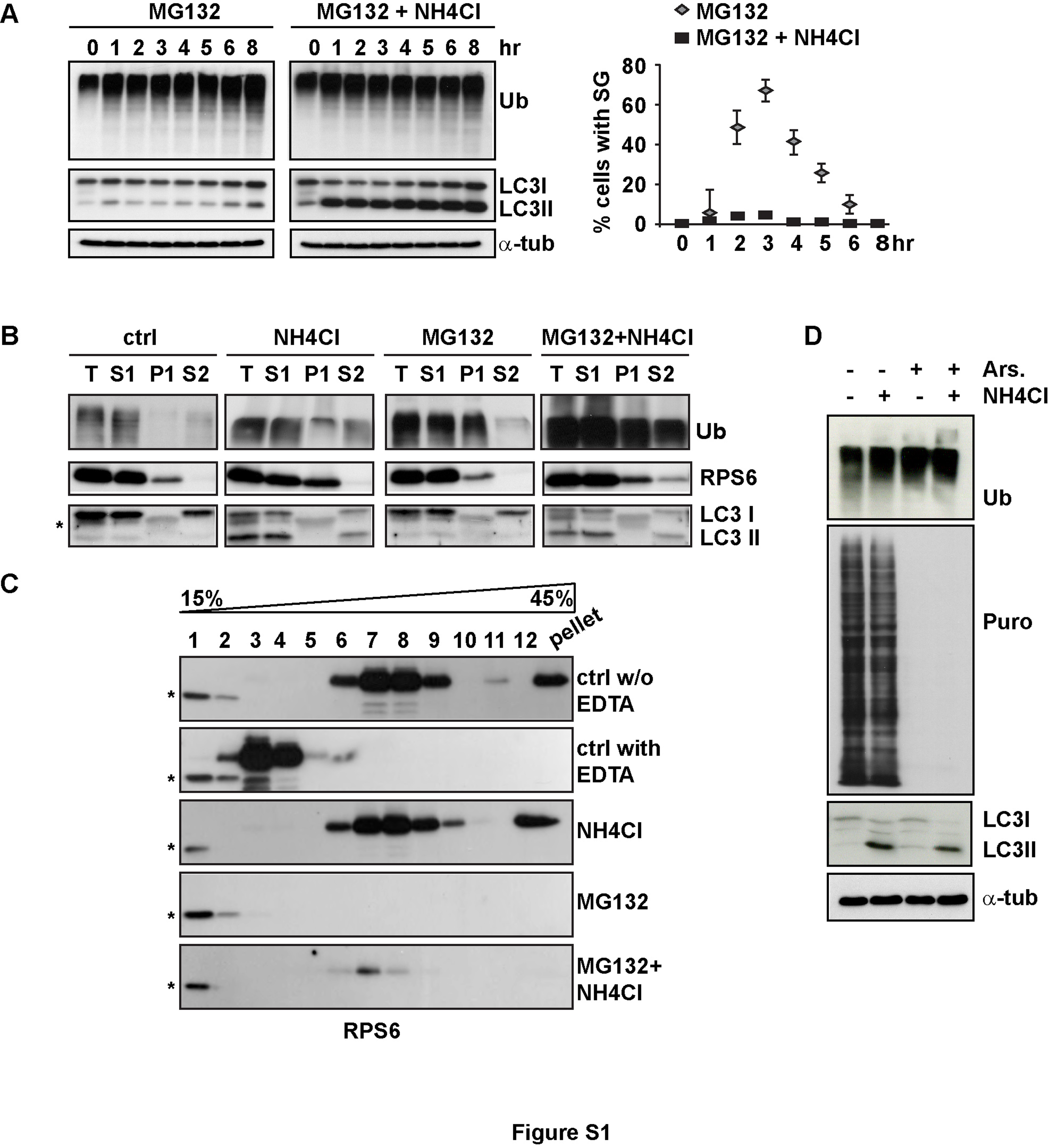

Supplement: Supplementary Figure S1 [file cdd2014103x2.tif]

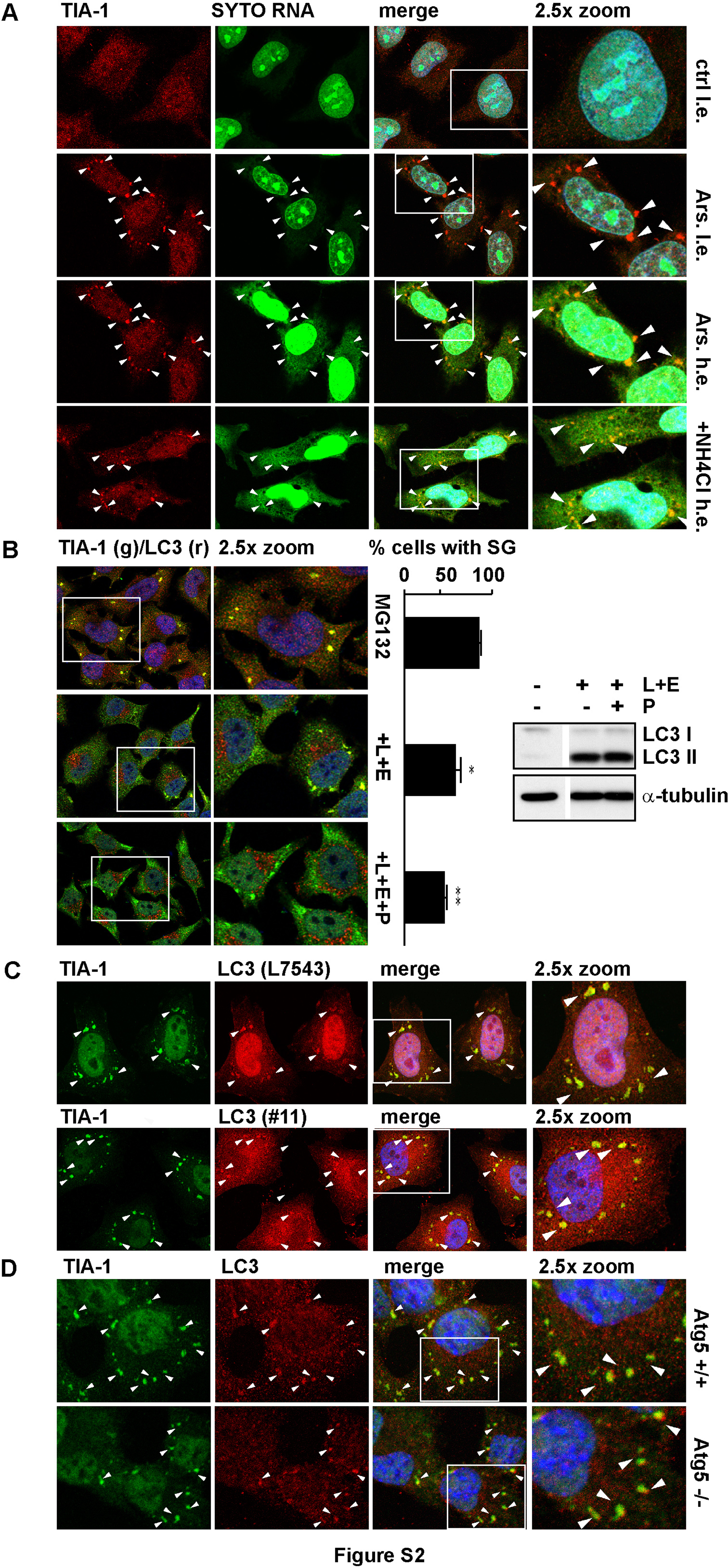

Supplement: Supplementary Figure S2 [file cdd2014103x3.tif]

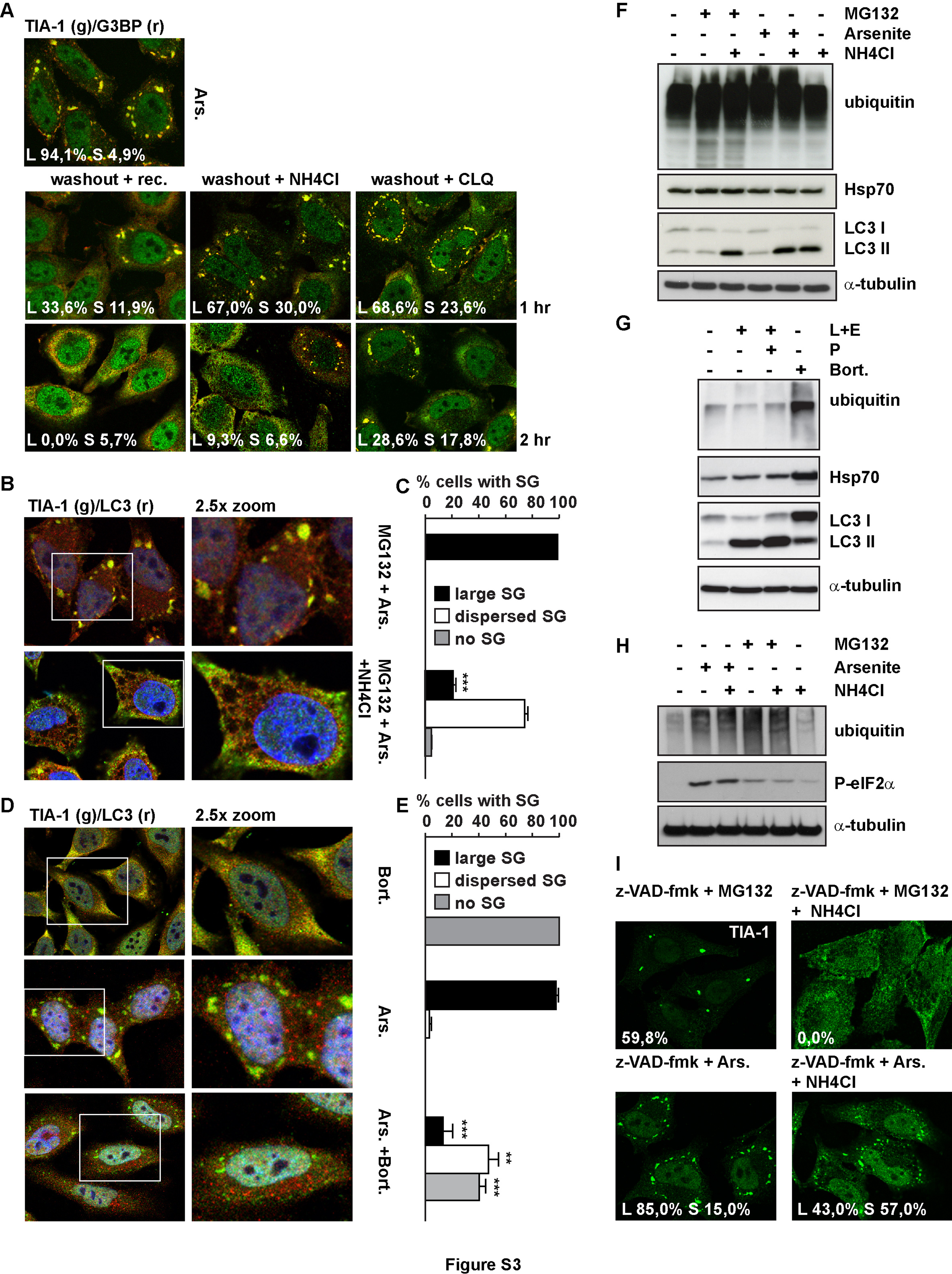

Supplement: Supplementary Figure S3 [file cdd2014103x4.tif]

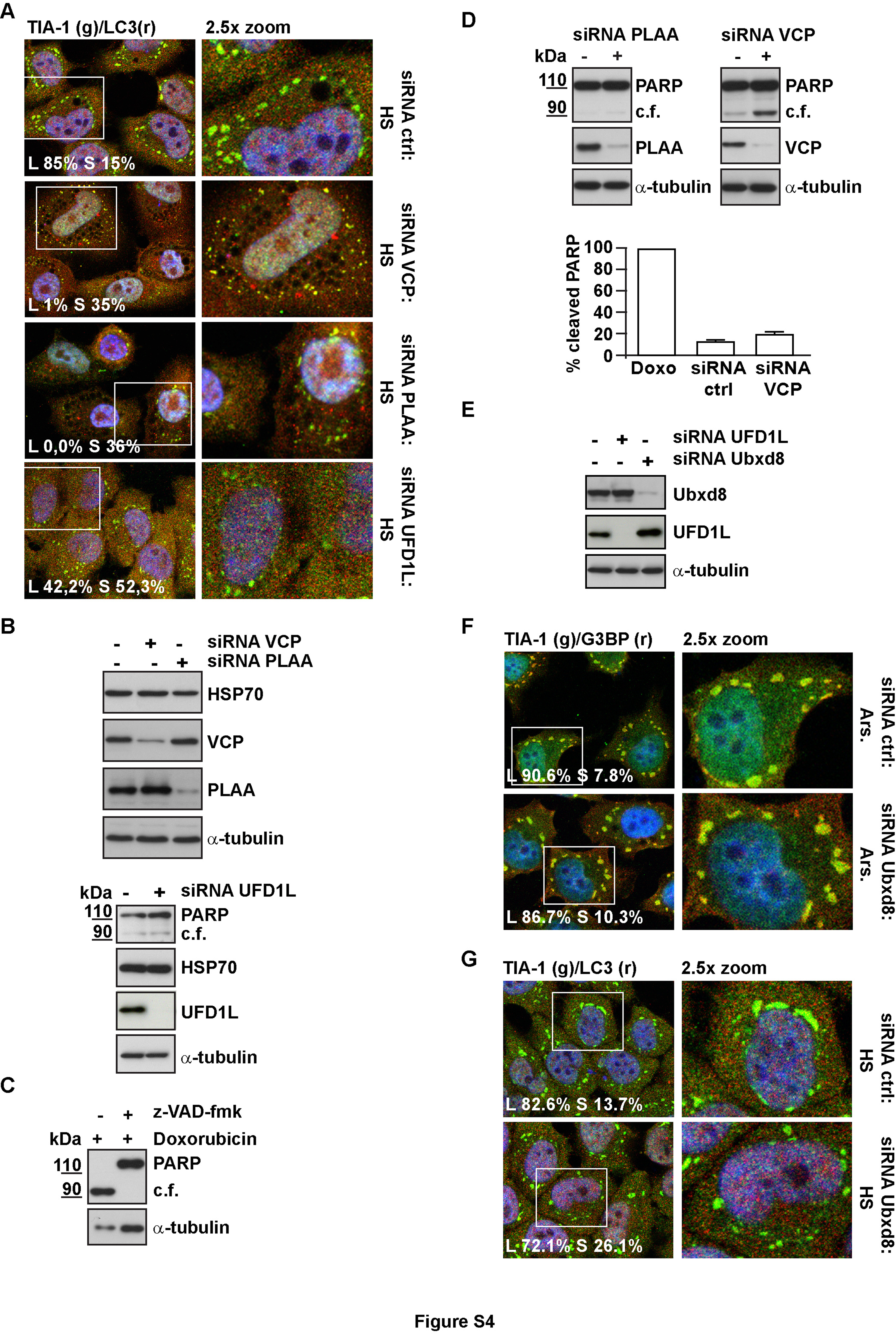

Supplement: Supplementary Figure S4 [file cdd2014103x5.tif]

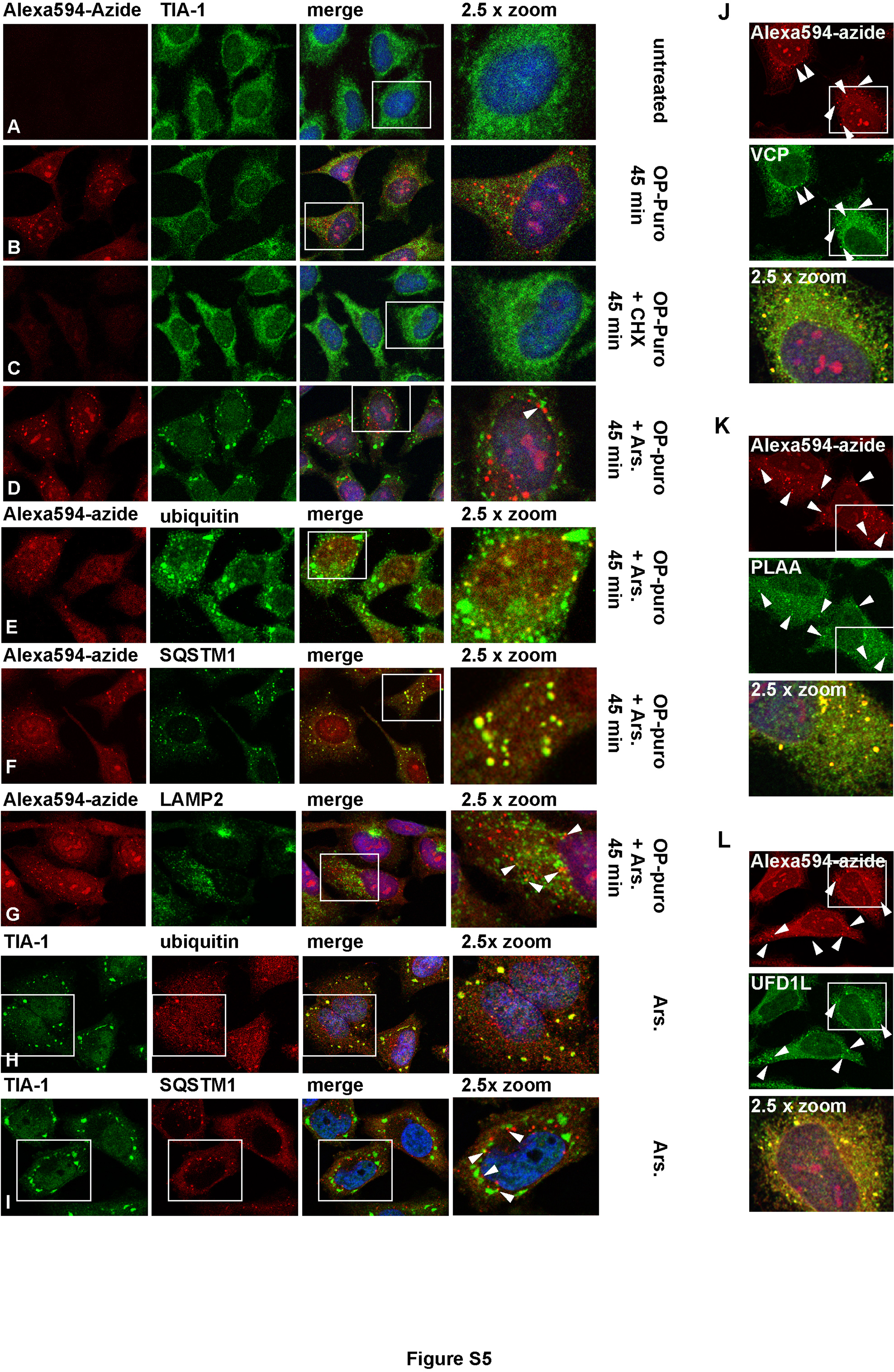

Supplement: Supplementary Figure S5 [file cdd2014103x6.tif]

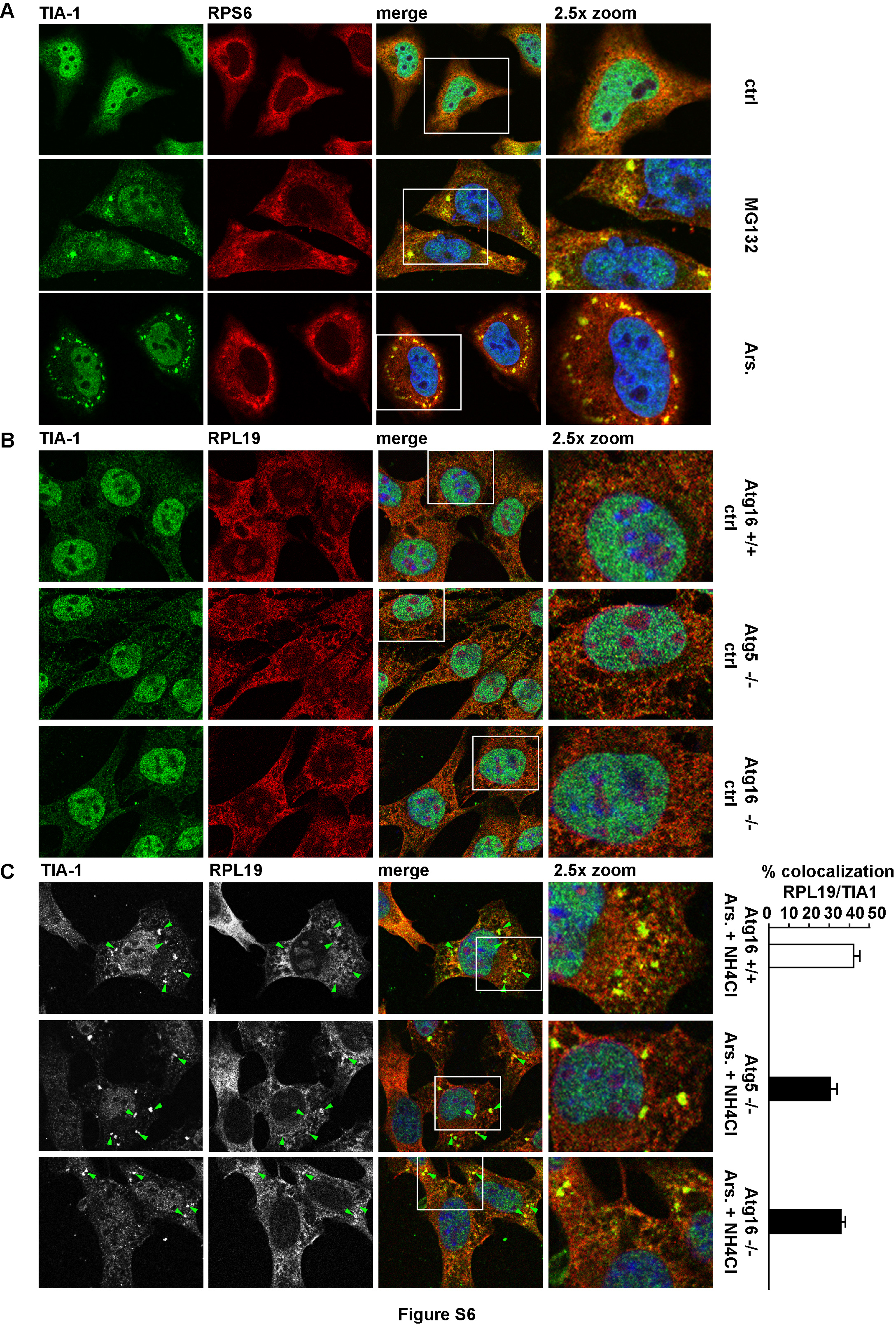

Supplement: Supplementary Figure S6 [file cdd2014103x7.tif]
