# Supplementary material for: Inhibition of autophagy, lysosome and VCP function impairs stress granule assembly
Source: Cell Death Differ. 2014 Jul 18;21(12):1838–51. doi: 10.1038/cdd.2014.103 (PMC4227144; doi:10.1038/cdd.2014.103)
Supplement: Supplementary Tables [file cdd2014103x8.pdf]

Supplementary Tables and Table Legends

Supplementary Table S1: % of necrotic (N) cells, apoptotic (A) cells and cells with depolarized mitochondria (DM) in HeLa cells treated with arsenite, lysosome and/or proteasome inhibitors.

HeLa cells were either left untreated (control) or treated with 20 µM MG132 for 3h, 20 mM NH4Cl for 3h or 0.5 mM arsenite for 45 min. Where indicated (+NH4Cl), cells were cotreated for 3h with MG132 and NH4Cl or pretreated for 2 hr 15 min with NH4Cl prior to the addition of arsenite. Mitochondria depolarization was induced by treating HeLa cells with 0.1 mg/ml valinomycin for 10 min. Cells were next processed for subgroup analysis by fluorescence-activated cell sorting (FACS) of apoptosis and cell death induction (measured by annexin V-FITC and Propidium iodide incorporation) and of mitochondria depolarization (measured by JC-1 incorporation). Representative percentages of necrotic cells (N), apoptotic cells (A) and cells with depolarized mitochondria (DM) are shown.

	**Hela cells**		
	**N**	**A**	**MD**
**Control**	3,88	7,63	5,33
**Arsenite**	5,47	10,57	6,69
**MG132**	10,5	11,31	4,19
**Arsenite+NH4Cl**	5,01	13,71	3,08
**MG132+NH4Cl**	4,81	14,55	6,72
**NH4Cl**	4,60	12,13	5,37
**Valinomycin**	na	na	94,4

Supplementary Table S2: % of necrotic (N), apoptotic (A) cells and cells with depolarized mitochondria (DM) in MEFs treated with arsenite, lysosome and/or proteasome inhibitors.

Autophagy proficient mouse embryonic fibroblasts (wildtype MEFs), Atg5 knockout MEFs and Atg16 knockout MEFs were either left untreated (control) or treated with 20 µM MG132 for 3h, 20 mM NH4Cl for 3h or 0.5 mM arsenite for 45 min. Where indicated (+NH4Cl), cells were cotreated for 3h with MG132 and NH4Cl or pretreated for 2 hr 15 min with NH4Cl prior to the addition of arsenite. Cells were next processed for subgroup analysis by fluorescence-activated cell sorting (FACS) of apoptosis and cell death induction (measured by annexin V-FITC and Propidium iodide incorporation) and of mitochondria depolarization (measured by JC-1 incorporation). Representative percentages of necrotic cells (N), apoptotic cells (A) and cells with depolarized mitochondria (DM) are shown.

	**wildtype MEFs**			**Atg5 knockout MEFs**			**Atg16 knockout MEFs**		
	N	A	DM	N	A	DM	N	A	DM
**Control**	6,09	10,04	6,77	3,47	8	1,64	1,97	8,11	1,07
**Arsenite**	1,81	5,02	1,42	1,5	4,2	0,14	3,08	5	0,65
**MG132**	11,20	13,3	2,75	12,40	11,53	0,67	1,73	9,38	1,59
**Arsenite+NH4Cl**	6,88	8,01	1,42	7,44	5,06	1,68	4,81	5,21	1,07
**MG132+NH4Cl**	6,14	10,39	2,96	0,79	10,57	1,26	10,40	14,08	3,8
**NH4Cl**	3,23	11,74	4,5	1,52	9,69	2,87	3,40	8,71	1,34
